# Regenerating the Pulp–Dentine Complex Using Autologous Platelet Concentrates: A Critical Appraisal of the Current Histological Evidence

**DOI:** 10.1007/s13770-020-00291-3

**Published:** 2020-11-04

**Authors:** Amna Riaz, Furqan A. Shah

**Affiliations:** 1 Department of Operative Dentistry, Pakistan Institute of Medical Sciences, Ibn-e-Sina Rd, G-8/3, Islamabad, Pakistan; 2 Department of Biomaterials, Sahlgrenska Academy, University of Gothenburg, 405 30 Gothenburg, Sweden

**Keywords:** Autologous platelet concentrate, Platelet-rich plasma, Platelet-rich fibrin, Regenerative endodontics

## Abstract

*****Background***:**

Autologous platelet concentrates such as platelet-rich plasma (PRP) and platelet-rich fibrin (PRF) have gained overwhelming popularity in regenerative endodontics. Clinical evidence reveals the lack of a particular advantage of using PRP or PRF over an evoked blood clot in promoting canal wall thickening and/or continued root development in immature necrotic teeth. Moreover, despite stimulating tissue repair and repopulating the root canals of immature and mature permanent teeth, the new vital tissue may not possess the functional activity of the native pulp tissue.

*****Methods***:**

To better understand the origin, nature, and long-term fate of the tissue types found within the pulp space, we critically examine all available histo-/morphological evidence for pulp–dentine complex regeneration using PRP and/or PRF, alone or together with an evoked blood clot, specialised or unspecialised primary cells, and other biomaterials.

*****Results***:**

Histological data from clinical studies is scant. Reportedly, the inner dentinal surface supports cementum-like tissue formation, but this interface likely deviates in structure and function from the native cementodentinal junction. Presence of bone-like tissue within the pulp space is intriguing since de novo osteogenesis requires closely coordinated recruitment and differentiation of osteoprogenitor cells. Compared to untreated necrotic teeth, an evoked blood clot (with/without PRF) improves fracture resistance. Tooth regeneration using PRF and dental bud cells is unreliable and the constituent neoformed tissues are poorly organised.

*****Conclusion***:**

PRP/PRF fail to demonstrate a significant advantage over an induced blood clot, alone. The true nature of neoformed tissues remains poorly characterised while their response to subsequent insult/injury is unexplored.

## Introduction

Regenerative endodontics has evolved over the years as a treatment option for not only immature permanent teeth with open apices (i.e., incomplete root development) but also for apical periodontitis in mature permanent teeth [1–3]. Based on the triad of adult stem/progenitor cells, morphogens, and a suitable scaffold, the focus of regenerative endodontics is to reconstitute the lost tooth tissue, i.e., the pulp–dentine complex [4]. Clinically and experimentally, one of the most frequently used technique comprises of intentionally provoked periapical bleeding in order to induce a blood clot within the disinfected canal. Rich in stem cells, inflammatory cells, fibroblasts, and growth factors that are essential to wound healing [5], evidence indicates that a blood clot act as a reliable tissue engineering scaffold in regenerative endodontics [6]. Since platelets are key players in wound healing pathways and release substances such as vascular endothelial growth factor (VEGF) and transforming growth factor beta (TGF-$\beta$) [7, 8], autologous platelet concentrates such as platelet-rich plasma (PRP) and platelet-rich fibrin (PRF) have received widespread attention in regenerative endodontics. PRP is rich in native growth factors, including platelet-derived growth factor, transforming growth factor, vascular endothelial growth factor, epithelial growth factor, and insulin like growth factor [9], and has been viewed as an alternative to an evoked blood clot [1]. PRF develops a fibrin network and the delivery of growth factors is continuous over time, however, degranulation of platelets and leukocytes implies a burst release of growth factors that promote cell proliferation and extracellular matrix remodelling [10].

In a systematic review of clinical studies, the lack of a particular advantage of using PRP or PRF compared to an evoked blood clot, alone, in promoting thickening of the canal walls and/or continued root development in immature necrotic teeth is strongly underscored [11]. Currently available clinical diagnostic methods do not provide information on tissue architecture, type and morphology of cells, and components of the extracellular matrix. Another systematic review of experimental studies concludes that PRP and PRF are able to stimulate tissue repair in both immature and mature permanent teeth, but despite repopulating the root canal, the new vital tissue may not possess the functional activity of the native pulp tissue [12]. In order to answer definitively if autologous platelet concentrates are a viable alternative to conventional endodontic procedures, this review aims to derive better understanding of the origin, nature, and long-term fate of the tissue types found within the pulp space (henceforth referred to as *neoformed* tissues).

## Review

Here, we critically examine the existing histological and morphological evidence for pulp–dentine complex regeneration using PRP and PRF, alone or in combination with an evoked blood clot, specialised or unspecialised primary cells, and other biomaterials. Using MEDLINE^®^/PubMed^®^, Scopus, Google Scholar, and ScienceDirect, the following search term (or equivalent) was applied: “platelet rich AND pulp”. All non-English language publications were disregarded. Publications that deviated from the theme (i.e., pulp–dentine complex regeneration), review papers, *in vitro* studies, and *in vivo* studies that did not report histological, morphological, or immunohistochemical data were excluded. Based on these criteria, a total of 21 publications were shortlisted (Table 1).

**Table 1:** Platelet-rich plasma (PRP), and platelet-rich fibrin (PRF) for pulp–dentine complex regeneration: summary of studies (in chronological order) reporting morphological or immunohistochemical data

Species	Teeth	Treatment groups	Experiment; scenario	Irrigation	Follow up	Observations	MTA^†^	References
Human; 12 y. o.	Premolar #4	PRP	Pulp necrosis and apical periodontitis	5.25% NaOCl; TAP	14 mo	Vital pulp-like connective tissue	Yes	[13, 14]
Rat; Wistar	Incisor	PRP EMD Ca(OH)_2_ MTA Ctrl^+^ Ctrl^−^	Pulp exposure + direct pulp capping	1% NaOCl	1 w 4 w	Reparative dentine (all groups); increase in Od-like cells (all except EMD)	No^‡^	[15]
Pig; miniature	Molar	FG FG + PRP FG + PRP + DBC	Autograft into alveolar socket		36 w	Odontogenesis (FG + PRP + DBC only); low success rate	No	[16]
Dog; Beagle	Premolar	BC DPSC PRP^Th^ DPSC + PRP^Th^	Pulpectomy	5.25% NaOCl; 17% EDTA	3 mo	New vital (bone-, cementum-, and PDL-like) tissues; no advantage of PRP^Th^ over BC	Yes	[17]
Dog; Beagle	Premolar	BC BC + PRP^Th^ BC + BMA BC + PRP^Th^ + BMA	Oral exposure induced pulp necrosis and apical periodontitis	2.5% NaOCl; 17% EDTA; TAP	3 mo	New vital (bone-, cementum-, and PDL-like) tissues; no advantage of PRP^Th^ and/or BMA^Th^ over BC	Yes	[18]
Human; 9 y.o.	Molar #30	BC BC + PRP	Pulp necrosis and apical periodontitis	5.25% NaOCl	25 mo	Mineralised (bone- and cementum-like) and fibrous tissues	Yes	[19]
Monkey	Incisor Canine	HAp HAp ± PRP	Pulpotomy High-level pulpotomy		6 mo	Lower root growth retardation, increased dentine bridge formation in HAp + PRP	No	[20]
Dog; Beagle	Premolar	BC BC + DPC PRP^Th^ PRP^Th^ + DPC	Plaque induced apical periodontitis	1.25% NaOCl; TAP	3 mo	More new vital tissues with PRP^Th^, more mineralised tissue with DPC, bone-like tissue in DPC + PRP^Th^	Yes	[21]
Ferret	Canine	BC PRP^Th^ Ctrl^+^	Pulpectomy	17% EDTA	3 mo	Comparable mineralised tissue formation for BC and PRP^Th^	Yes	[22]
Dog; Beagle	Premolar	BC PRP	Plaque induced apical periodontitis	3% NaOCl; TAP	3 mo	Mineralised- and pulp-like tissue formation, and apical closure comparable for BC and PRP	Yes	[23]
Dog; Beagle	Premolar	BC DPSC PRP^Th^ DPSC + PRP^Th^	Pulpectomy	5.25% NaOCl; 17% EDTA	3 mo	Cellular islands of mineral stain positive for BSP, OCN, TRAP, and periostin	Yes	[24]
Ferret	Canine	BC/Gelfoam^®^ PRP^Th^ Ctrl^+^ Ctrl^−^	Pulpectomy + induced necrosis	1% NaOCl; 17% EDTA; TAP	3 mo	Comparable mineralised tissue formation for BC/Gelfoam^®^ and PRP^Th^	Yes	[25]
Dog	Premolar	BC PRP^Th^ Ctrl^+^ Ctrl^−^	Plaque induced necrosis	5.25% NaOCl; TAP	1 mo 3 mo	Comparable amounts of new vital connective tissue for BC and PRP^Th^	Yes	[26]
Dog; Beagle	Premolar	BC PRP TAP^m^ + BC TAP^m^ + PRP Ctrl^+^ Ctrl^−^	Plaque induced necrosis and apical periodontitis	1.25% NaOCl; 17% EDTA ± TAP^m^	6 mo	Highest mineralised tissue deposition on dentinal walls and apical closure for TAP^m^ + PRP	Yes	[27]
Dog; Beagle	Premolar	PRF Empty canal Ctrl^+^	Pulpectomy	5.25% NaOCl; 17% EDTA	12 w	150% greater root wall thickness with PRF	Yes	[28]
Dog; Mongrel	Premolar	BC BC + PRP^Th^ BC + Parafilm^®^	Oral exposure induced pulp necrosis	NaOCl; TAP	3 mo	New vital connective-, and cementum- and bone-like tissues; no advantage of PRP^Th^	Yes	[29]
Dog; Beagle	Premolar	BC BC + PRF Ctrl^−^	Plaque induced apical periodontitis	1.25% NaOCl; 17% EDTA; TAP	3 mo	Comparable new cementum- and bone-like tissue comparable for BC and BC + PRF	Yes	[30]
Human; 29 y.o.	Incisor #7	PRF	Previous trauma, pulp necrosis and apical periodontitis	3% NaOCl; 17% EDTA	6 mo 12 mo	Acellular cementum-like tissue externally with diffuse calcification associated with dentinal wall	Yes	[31]
Dog	Premolar	PRF Ca(OH)_2_	Pulpectomy ± oral exposure induced pulp necrosis and apical periodontitis	Ca(OH)_2_; 0.9% NaCl	6 mo	More mineralised tissue deposition on dentinal walls for PRF in contaminated teeth	Yes^††^	[32]
Dog; Beagle	Premolar Molar	BC PRP Collagen sponge	Inflammatory plug induced by over-instrumentation	0.5% NaOCl; 17% EDTA	8 w	Apical inflammatory plug, root resorption, lack of cellular infiltration or regeneration	Yes	[33]

^Th^ = added thrombin; # = Universal numbering system; ^†^ = MTA placed directly over PRP or PRF; ^††^ = Portland cement; ^‡^ = polymer-reinforced zinc oxide eugenol as restorative material; BC = blood clot evoked by over-instrumentation into apical tissues; BMA = bone marrow aspirate; BSP = bone sialoprotein; Ca(OH)_2_ = calcium hydroxide; Ctrl^−^ = untreated necrotic/infected pulp OR no ‘experimental’ biomaterial used; Ctrl^+^ = normal/unaffected tooth OR no cavity preparation; DBC = dental bud cells; DPC = dental pulp cells; DPSC = dental pulp stem cells; EDTA = ethylenediaminetetraacetic acid; EMD = enamel matrix derivative; FG = fibrin glue; Gelfoam^®^ = resorbable gelatin sponge; HAp = hydroxy(l)apatite; mo = month(s); MTA = mineral trioxide aggregate; NaOCl = sodium hypochlorite; OCN = osteocalcin; Od = odontoblast; PDL = periodontal ligament; PRF = platelet-rich fibrin; PRP = platelet-rich plasma; TAP = triple antibiotic paste (metronidazole, minocycline, and ciprofloxacin); TAP^m^ = modified triple antibiotic paste (metronidazole, cefixime, and ciprofloxacin); TRAP = tartrate-resistant acid phosphatase; w = week(s)

**Fig. 1:**
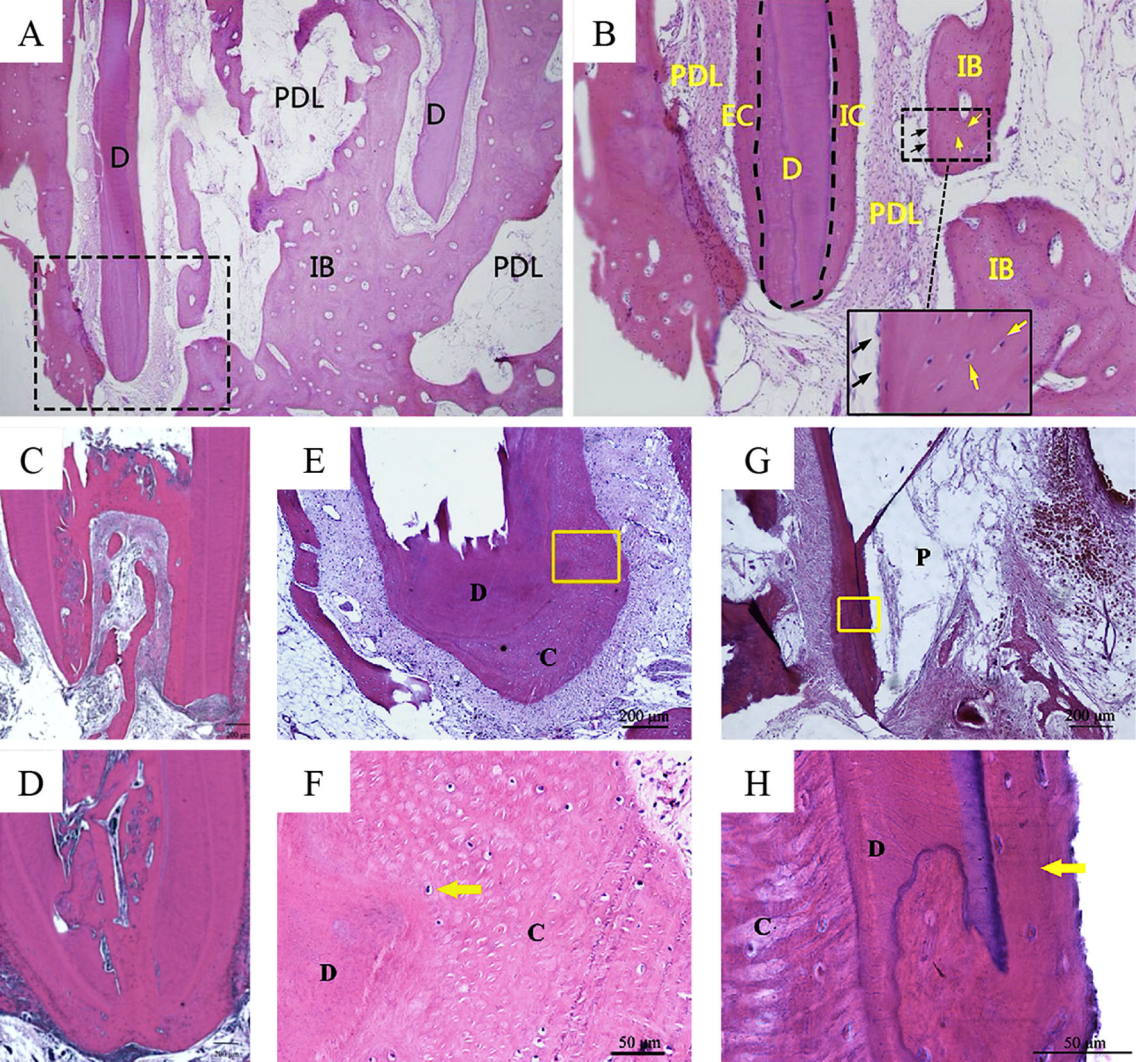
Neoformed vital tissues. **A**, **B** Osteoblast-like cells (black arrows) and osteocyte-like cells (yellow-arrows) associated with bone-like tissue within the canal space. D = dentine, EC = extracanal cementum, IB = intracanal bone, IC = intracanal cementum, PDL = periodontal ligament. From Zhou et al. [30]. J Endod. Adapted with permission from Elsevier. Copyright 2017 [30]. **C**, **D** Apical narrowing and closure. Narrowing of the apex by the ingrowth of bone-like tissue into the canals (**C**) and complete occlusion of the apex as bone-like and cementum-like tissues merge together (**D**). From Zhu et al. [21]. Int Endod J. Adapted with permission from John Wiley and Sons. Copyright 2013 [21]. **E**, **F** Upon apical closure, neoformed intrapulpal tissues in the canal space do not communicate with the periapical tissues. Cementocyte-like cell (arrow), C = cementum, D = dentine. **G**, **H** Mineralised tissue deposition along the inner canal wall. Cellular cementum-like tissue (arrow), C = cementum, D = dentine, P = pulp. Hematoxylin–Eosin staining in **A**–**H**. From Zhang et al. [23]. J Endod. Adapted with permission from Elsevier. Copyright 2014 [23]

Briefly, studies report the application of autologous platelet concentrates across a range of clinically relevant scenarios. The parameters typically evaluated include presence of various relevant cell types (e.g., odontoblasts, cementoblasts, osteoblasts, fibroblasts), continued root development (or root lengthening), narrowing or closure of the root apex, formation of reparative dentine, presence of neoformed vital (mineralised and unmineralised) tissues, ingrowth of bone-like and cementum-like tissues, presence of islands of bone-like and cementum-like tissues, deposition of mineralised tissue on the inner canal/dentinal wall, thickening of the canal wall, and presence/absence of inflammatory cells (Fig. 1).

### Experimental studies

#### Pulp exposure, pulpotomy, and pulpectomy

Among the most basic endodontic applications of platelet-rich plasma (PRP) and platelet-rich fibrin (PRF) are iatrogenic pulp exposure, pulpotomy in immature teeth, and pulpectomy in mature teeth. For direct pulp capping, a protective, circumferential layer of reparative dentine along the root canal walls can be achieved with PRP, Ca(OH)_2_, mineral trioxide aggregate (MTA), and enamel matrix derivative (EMD). However, in terms of reparative dentine thickness and odontoblast density, the use of PRP does not offer a significant advantage for intracanal tissue regeneration [15].

In partial- and high-level pulpotomy procedures where hydroxy(l)apatite (HAp) is used in combination with PRP, root growth retardation and reparative dentine bridge formation occur as frequently as when HAp is used alone. It is noteworthy that, regardless of therapeutic modality, high-level pulpotomy tends to afford a less favourable outcome than partial pulpotomy [20].

Pulpectomy entails complete extirpation of the pulp. The canal is subsequently irrigated and dried using sterile paper points. Using PRP, ingrowth of bone-like tissue is observed throughout the canal—from the coronal third to the apex of the root. Such islands of bone-like tissue are dispersed between neoformed connective tissue comprised of blood vessels and fibroblasts, and populated with osteoblasts in addition to some osteoclasts on their surface. Canal walls tend to be thinner than normal teeth and there is no physiological narrowing of the apex through deposition of new dentine [22].

Intriguingly, the histological picture is no different when PRF is used. The neoformed vital tissues also include islands of bone-like tissue scattered between connective tissue made up of blood vessels and fibroblasts. In addition, dentine-like tissue exhibiting an irregular tubular structure forms along the inner dentinal wall but apparently discontinuous from the native dentinal tubules. Not surprisingly, odontoblasts associated with this dentine-like tissue do not display the typical polarised and palisade appearance [28].

**Fig. 2:**
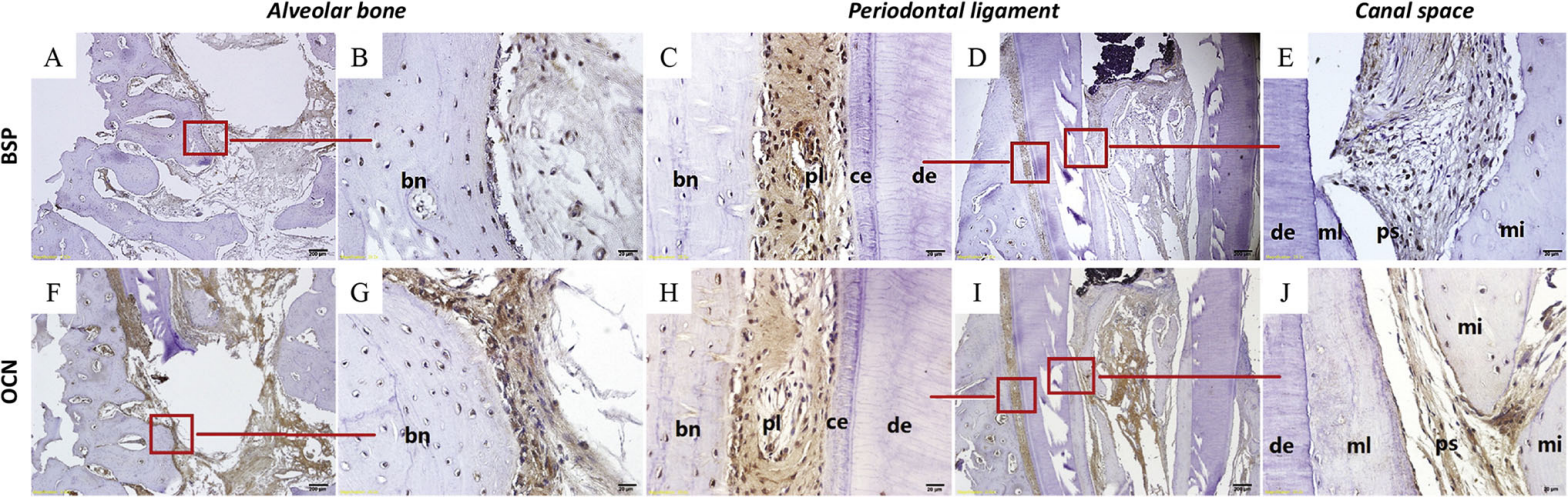
Bone sialoprotein (BSP) and Osteocalcin (OCN) in the alveolar bone, periodontal ligament, and canal space. **A**–**E** Cells embedded within the alveolar bone, periodontal ligament, and fibrous tissue in the canal space stain positive for BSP. **F**–**J** Cells surrounding the alveolar bone, periodontal ligament, and fibrous tissue in the canal space stain positive for OCN. bn = bone, ce = cementum, de = dentin, mi = newly formed mineral island, ml = newly formed mineral lamina (along inner canal/dentinal wall), pl = periodontal ligament, ps = pulp space. From Zhu et al. [24]. J Endod. Adapted with permission from Elsevier. Copyright 2014 [24]

Even in combination with dental pulp stem cells (DPSCs), PRP shows no enhancement in new tissue formation compared to an evoked blood clot into the root canals [17]. The incidence of full-length (above the cervical level up to the pulp horns) and half-length (cessation at mid root level) regeneration is comparable for PRP with or without DPSCs. Reportedly, half-length regeneration is attributable to the presence of inflammatory cells coronal to the regenerated tissue. Ingrowth of cementum-like tissue is seen along the inner surface of the canal wall, and appears to be continuous with the outer root surface. This cementum-like tissue is thickest at the apex and progressively thinner in the coronal direction. Cementoblast-like cells can be identified. Fibrillar structures similar to Sharpey’s fibres insert into the cementum-like tissue. However, no dentine-like tissue is detected. In the coronal part of the pulp space, islands of bone-like tissue are noted in close spatial association with MTA [17]. These islands of bone-like tissue stain positively for bone sialoprotein and osteocalcin (Fig. 2), while a single layer of periostin-positive cells surrounding these islands is indicative of periosteum, in addition to tartrate-resistant acid phosphatase positive multinucleated cells adjacent to the islands of bone-like tissue [24].

#### Induced pulp necrosis and periapical inflammation

Experimentally, pulp necrosis and apical periodontitis can be induced by intentionally exposing the pulp to the oral environment or by placing a suspension of oral plaque within the pulp for an extended period of time. In necrotic teeth, the outcome of PRP is largely unremarkable compared to that of an evoked blood clot. The incidence of vital tissue regeneration and severity of inflammation are generally comparable [29]. The periapical lesion resolves [25], but dentine development is arrested [27] and the presence of an inflammatory cell infiltration is noted occasionally [23, 25]. Picrosirius red staining and polarised optical microscopy confirm the presence of collagen fibres within the neoformed bone-like, cementum-like, and connective tissue. Fibrillar structures similar to Sharpey’s fibres appear to anchor the connective tissue into the cementum-like tissue [18]. The new connective tissue contains many blood vessels and fibroblasts [18, 23, 25]. Expression of VEGF and factor VIII of the coagulation cascade by the stromal and endothelial cells of blood vessel walls is initially high but gradually diminishes [26]. Distributed throughout the canal space, islands of bone-like tissue are also identified, with matrix-bound osteocytes and osteoblasts and osteoclasts at the surface [18, 25, 27]. Cementum-like tissue, containing cells similar to cementoblasts at the surface and cementocytes distributed within the bulk, lines the dentinal wall [18]. Furthermore, islands of cellular cementum-like tissue are also detected within the canal space. But despite apical closure, the regenerated connective tissue within the root canal, in a high proportion of cases, does not reconnect with the periodontal ligament at the apex [23]. Despite thickening of the root wall in the apical third attributable to the ingrowth of bone-like and cementum-like tissues along the inner surface of the canal wall [25, 27], however, root lengthening does not occur [25].

**Fig. 3:**
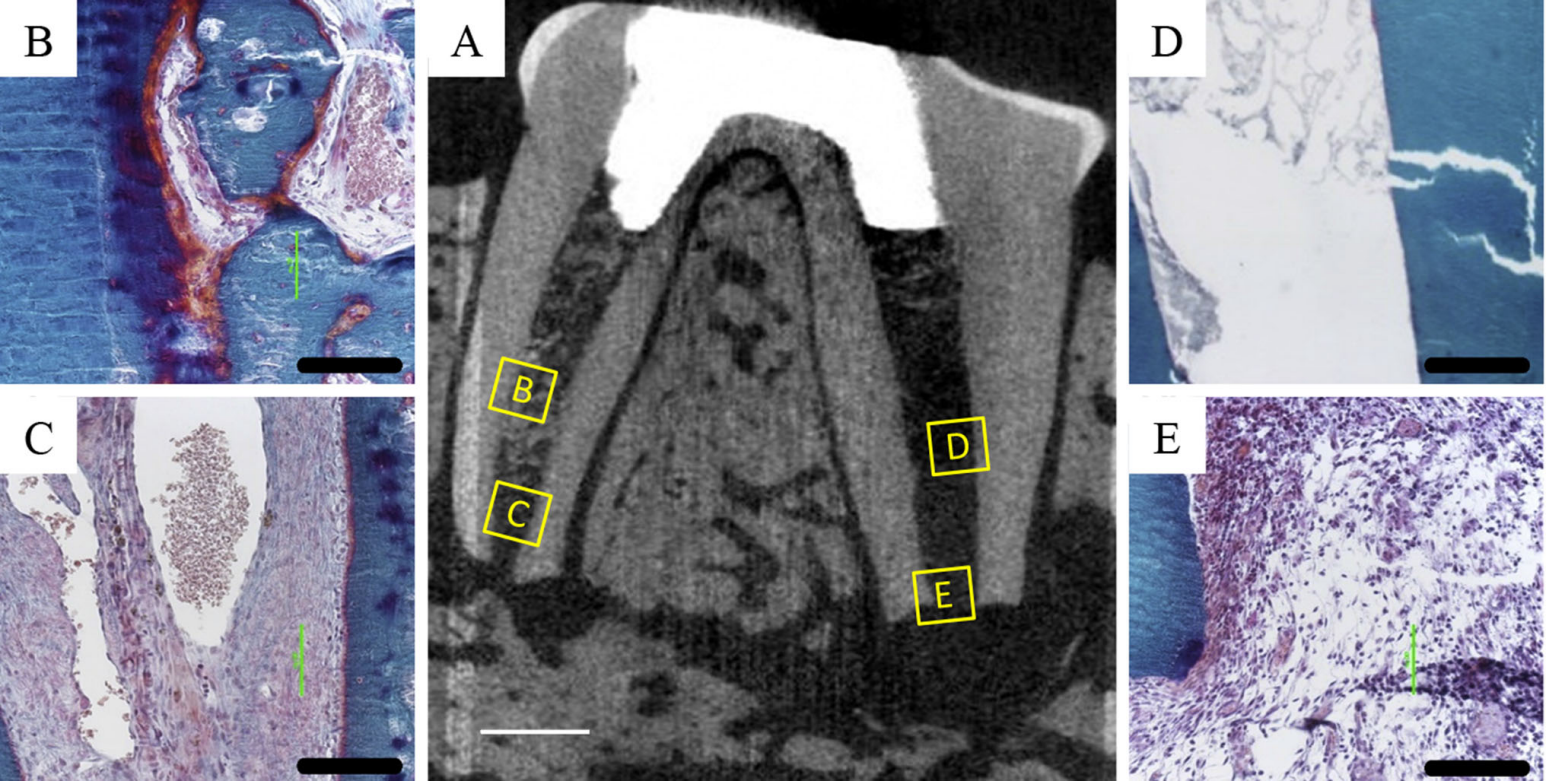
Cellular infiltration and tissue formation in premolars 12 weeks after root canal manipulation and PRF placement. **A** X-ray micro-computed tomography. **B**, **C** Roots manipulated at the apex show intact roots and periodontal ligament, intraradicular cellular infiltration and regeneration, and no signs of periapical inflammation. **D**, **E** Roots manipulated beyond the apex show root resorption, no intraradicular cellular infiltration, and signs of periapical inflammation. Goldner’s trichrome staining in **B**–**E**. Scale bars: A = 2 mm; B–E = 100 μm. From Zaky et al. [33]. J Endod. Adapted with permission from Elsevier. Copyright 2020 [33]

Canal disinfection using a preparation containing metronidazole, cefixime, and ciprofloxacin in equal parts significantly increases the occurrence of apical closure and mineralised tissue deposition on dentinal walls in response to PRP [27]. Canal irrigation and disinfection using only 0.9% NaCl and Ca(OH)_2_ paste does not lead to an improvement in the efficacy of PRF compared to that in aseptically extirpated pulps [32]. However, PRF exhibits a higher incidence of vital tissue formation in aseptic canals and outperforms Ca(OH)_2_ in its ability to support mineralised tissue deposition on dentinal walls of previously contaminated canals [32]. Periapical inflammation, achieved experimentally by aseptic instrumentation beyond the root apex, leads to a dense accumulation of inflammatory cells including M1 macrophages (identified as CD86-positive cells) at the apex that occlude the canal entrance and act as a barrier against cellular infiltration into the root canal (Fig. 3). Even with the use of PRP, this barrier, or “inflammatory plug” is associated with root resorption and lack of vital tissue regeneration [33].

An evoked blood clot, both alone and together with PRF, facilitates resolution of plaque induced apical periodontitis. Radiographic measurements indicate greater increases in root length and thickness, while histological observations reveal very high incidence of neoformed mineralised tissue, characterised as islands of bone-like tissue dispersed within the canal space and cementum-like tissue at the apex and along the inner surface of the canal wall [30]. Furthermore, application of compressive loads at an angle of 45° to the long axis of the tooth suggest greater resistance to fracture with (~ 281.7 N) and without (~ 249.3 N) PRF compared to untreated necrotic teeth (~ 108.5 N).

One study reports an increase in the area fraction of canal space occupied by vital tissues with the use of PRP, either alone or in combination with dental pulp cells (DPCs), compared to an evoked blood clot. The neoformed tissues comprised of fibrous connective tissue and vasculature, in addition to cementum-like and bone-like tissues. Interestingly, the fraction of unmineralised tissue in response to PRP with DPCs was only 30% that of PRP without DPCs, suggesting the potential of DPCs to stimulate mineralised tissue formation [21]. The combination of DPCs and PRP was associated with occlusion of the apex by coalescence of neoformed cementum-like and bone-like tissues. Avascular, cementum-like tissue grew along the inner canal wall via the apex, while bone-like tissue appeared in the form of mineralised islands that were both cellular and vascular. However, only 35% of the canals treated using PRP contained neoformed tissues reaching up to the coronal third.

#### Tooth regeneration

**Fig. 4:**
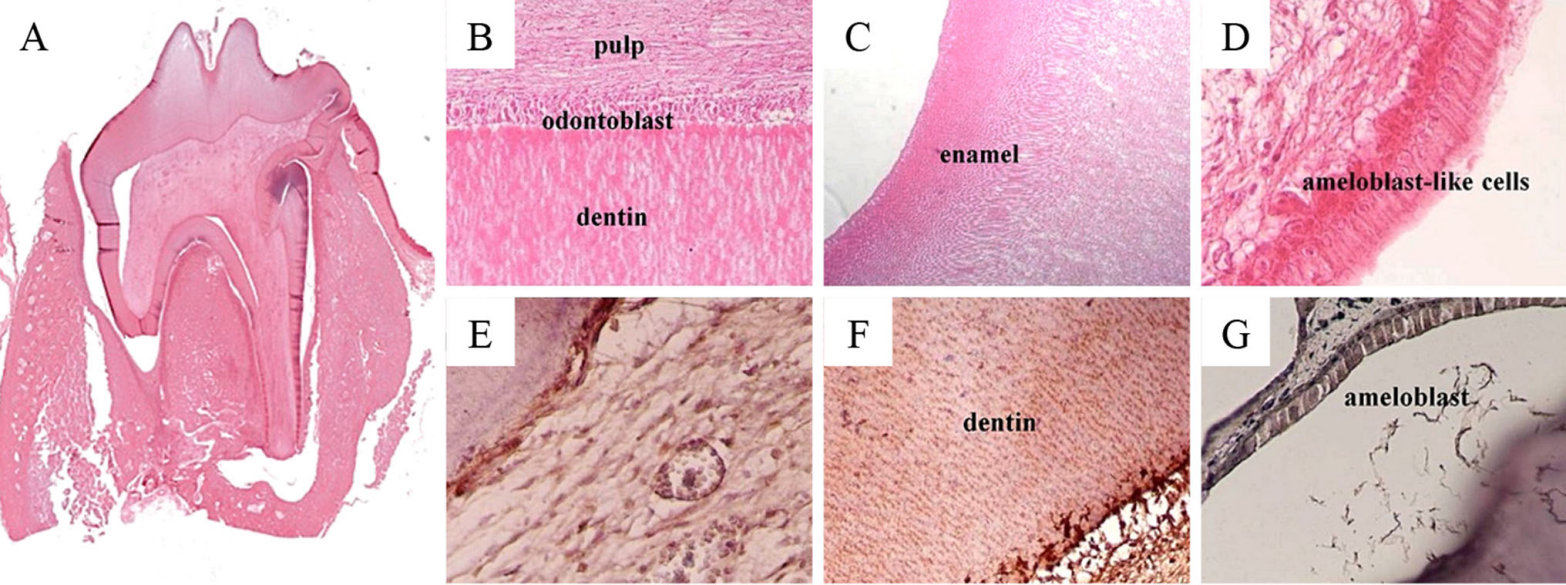
Tooth regeneration—histology and immunohistochemistry. **A** The regenerated tissues include alveolar bone, dentine, and connective tissue. **B** Dentine, odontoblasts, and pulp. **C** Enamel. **D** Ameloblast-like cells. **E** Vascular Endothelial Growth Factor (VEGF) expression in blood vessel endothelial cells of the dental pulp. **F** Dentine Matrix Protein-1 (DMP-1) expression in dentine. **G** Cytokeratin 14 (CK14) expression in ameloblasts. Hematoxylin–Eosin staining in **A**–**D**. From Yang et al. [16]. J Tissue Eng Regen Med. Adapted with permission from John Wiley and Sons. Copyright 2012 [16]

The possibility to regenerate an entire tooth has been demonstrated using a PRF and fibrin glue scaffold seeded with dental bud cells, which was subsequently implanted into the alveolar socket [16]. Tooth regeneration could be achieved in two out of nine implantation sites, of which only one was able to erupt into the oral cavity. Although the regenerated teeth contained all requisite tissue types (i.e., enamel, dentine, cementum, alveolar bone, and connective tissue) identified using immunohistochemical analyses, the anatomy did not resemble that of a ‘normal’ tooth (Fig. 4).

### Clinical studies

Among clinical studies, one instance of a necrotic pulp and immature/open apex of a premolar tooth that was accidentally extracted and immediately replanted, radiographic examination revealed evidence of continued root development following the use of PRP [13]. The regenerated tissues were, however, removed with a barbed broach at 14 months and processed for histological examination, which revealed the presence of vital pulp-like connective tissue containing fibroblasts, blood vessels, and a few lymphocytes. While no odontoblasts were identified, a few flattened multinucleated foreign body giant cells surrounded granular basophilic material [14].

**Fig. 5:**
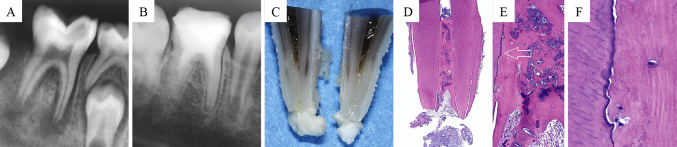
Platelet-rich plasma in a human tooth. **A** Preoperative radiograph. **B** Radiograph at 25-month follow-up after PRP placement. **C** Root divided longitudinally. **D** Histological section through the centre of the root canal. **E** Apical portion of the canal. **F** Cementoid/osteoid tissue along the root canal wall (at the level indicated by the arrow in **E**). Cementocyte-like or osteocyte-like cells reside within lacunae in the newly formed mineralised tissue. Hematoxylin–Eosin staining in **D**–**F**. From Martin et al. [19]. J Endod. Adapted with permission from Elsevier. Copyright 2013 [19]

In a molar tooth presenting with a large carious lesion, periapical radiolucent areas, and apical resorption, an evoked blood clot with PRP (in distal canals) or without PRP (in mesial canals) resulted in obliteration of the canal space by cementoid- or osteoid-like mineralised tissue and fibrous connective tissue (Fig. 5). However, no odontoblasts were identified [19]. Similarly, the use of PRF in a lateral incisor with a necrotic pulp, an immature/open apex, and apical periodontitis resulted in areas of diffuse mineralisation associated with the dentinal wall and acellular cementum-like tissue externally [31].

## Current challenges, concerns, and shortcomings in the available evidence

It is hardly surprising that the histological data reported by presumably successful clinical studies using autologous platelet concentrates for pulp–dentine complex regeneration is scant. The observations are contradictory and the evidence in favour of using platelet-rich plasma (PRP) and platelet-rich fibrin (PRF), therefore, remains less than persuasive. With the exception of one study exploring the efficacy of PRP against an induced inflammatory plug [33], no studies (whether experimental or clinical) report on the response of neoformed tissues to insult or injury. For example, if there is development of a structure analogous to reparative dentine. However, for such to occur, several functionally distinct cell types will have to function together in a near-native spatial arrangement. Although there is some evidence of revascularisation, the subject of innervation has largely been overlooked.

Which tissue type(s) should ideally occupy the pulp space after regenerative procedures? This question is not a new one [34]. However, very little is understood about the true nature of the tissue(s) occupying the pulp space following a therapeutic strategy aimed at regeneration or revitalisation. The appearance of neoformed dentine or even cementum within the pulp space may be viewed favourably—it cannot be denied that formation of a hard tissue bridge coronal to neoformed soft tissue is a desirable outcome. The presence of bone-like tissue within the pulp space is, however, liable to raise a number of questions. It is interesting that the inner dentinal surface supports the formation of cementum-like tissue, giving rise to an interface that likely deviates dramatically in structure, and therefore function, from the native cementodentinal junction. The presence of islands of neoformed bone-like tissue within the intrapulpal space, however, is worth some deliberation, as not only does de novo osteogenesis require recruitment but also differentiation of osteoprogenitor cells into osteoblasts and mineral-bound osteocytes, terminally. Furthermore, does this neoformed bone-like tissue undergo remodelling in response to mechanical loading experienced by the tooth? Indeed, adaptive remodelling of bone is known to occur within the confines of macro-porous bone anchored implants where newly formed bone is located remote from the native bone [35].

Limited attempts have been made to characterise the composition (e.g., inorganic and organic constituents and their relative quantities) or structure (e.g., micro- to nanometre level arrangement) of the extracellular matrix in neoformed mineralised tissues. Many of these questions can be addressed using analytical tools commonly applicable for characterisation of bone [36], including Raman spectroscopy [37], scanning electron microscopy [38], and transmission electron microscopy [39].

Currently, the longest follow up durations among experimental and clinical studies are 6- and 25 months, respectively. In a simian model of pulpotomy [20], the prevailing reparative capacity of the remaining viable pulp cells is highlighted by the more favourable outcome of partial pulpotomy compared to high-level pulpotomy, regardless of PRP use. In a canine model of plaque induced pulp necrosis and apical periodontitis [27], the efficacy of PRP in terms of incidence of apical closure and mineralised tissue deposition on dentinal walls appears to be closely linked to the extent of canal disinfection [27]. But in a human molar presenting with pulp necrosis and apical periodontitis, the use of PRP appeared to neither amplify nor diminish the presence of neoformed cementoid-/osteoid-like tissue and fibrous connective tissue, compared to an evoked blood clot alone [19].

Nevertheless, the presence of ectopic mineralised tissue brings up questions regarding long-term fate and consequences for functioning of the local environment. What factors modulate the formation of neoformed mineralised tissues? How does their architecture compare to the native tissues they appear to resemble? Would disproportionate amounts of neoformed mineralised tissue occlude the apex, as has been reported in the case of the combined application of PRP and dental pulp cells [21], and result in irreversible discontinuity between intracanal and periapical environments? These queries currently remain unaddressed. Do ectopic, and presumably vital, mineralised tissues provide reinforcement to the tooth structure (i.e., resistance to fracture), which has suffered irreversible embrittlement from the restorative/endodontic procedure(s)? Only one study offers some insight into this particular dilemma [30], where the resistance to fracture was shown to increase by ~ 130–160% when the treatment modality involved an evoked blood clot, either alone or in combination with PRF.

It is noteworthy that, without exception, all studies reporting the presence of neoformed bone-like tissue have used mineral trioxide aggregate (MTA) as the pulp capping material, which is composed of Portland cement as the major component [40, 41], bismuth oxide, and small additions of SiO_2_, CaO, MgO, K_2_SO_4_, and Na_2_SO_4_. This trend is unsurprising since as many as 85% of all clinical regenerative endodontic procedures use MTA as the coronal barrier [42]. Other calcium silicate-based materials such as Biodentine^®^, claimed to be similar in composition and comparable in efficacy to MTA [43], have also been used in combination with PRP [44] and PRF [45], clinically, for the treatment of immature teeth with necrotic pulps. However, in the absence of credible histo-/morphological evidence, clinical *success* perceived from radiographic observation of continued root development, apical closure, and resolution of periapical lesions must not be attributed to the use of an autologous platelet concentrate. The *in vitro* osteogenic potential [46] and *in vivo* bone-bonding ability [47] of MTA are well-established. To exclude the role of MTA and isolate the role of autologous platelet concentrate(s), an alternative biomaterial or composition that offers a comparable protective effect without inducing osteogenesis must be considered.

A common feature among descriptions of the various neoformed tissues is the morphological likeness attributed to different cell types identified. Examples include osteoblast-like, osteocyte-like, cementoblast-like, cementocyte-like, odontoblast-like, and fibroblast-like cells. It is unclear whether such terms serve to imply similarities or deviations in function compared to the corresponding cell types in native tissues.

The available quantitative data also presents shortcomings: discrete data (e.g., percentage of canals or teeth that contain a given tissue type) is less precise than continuous data (e.g., what fraction of each canal is occupied by a given tissue type on average). For this reason, it is highly probable that differences (or similarities) between the various treatment groups may have been lost to detection.

## Conclusion

Given the dearth of convincing data, the application of autologous platelet concentrates such as PRP or PRF in pulp–dentine complex regeneration does not appear to offer a significant advantage over an induced blood clot, alone. Many key questions arise and remain unanswered. It is apparent that the true nature of neoformed tissues, as reported by experimental and clinical studies employing autologous platelet concentrates, is far from the native tissue types and remains poorly characterised. In fact, it is highly likely that the types and relative proportions of neoformed tissues are primarily a function of canal preparation procedures. But to describe these as having *formed in response to* autologous platelet concentrates is strongly misleading.
